# Prevention and Control of Non-communicable Diseases in Iranian Population: Life Style Promotion Project Phase II: Study Protocol

**Published:** 2018-09

**Authors:** Jafar Sadegh TABRIZI, Mostafa FARAHBAKHSH, Homayoun SADEGHI-BAZARGANI, Leila NIKNIAZ

**Affiliations:** 1. Tabriz Health Services Management Research Center, Faculty of Management and Medical Informatics, Tabriz University of Medical Sciences, Tabriz, Iran; 2. Research Center of Psychiatry and Behavioral Sciences, Tabriz University of Medical Sciences, Tabriz, Iran; 3. Road and Traffic Injury Research Center, Dept. of Statistics and Epidemiology, Faculty of Health, Tabriz University of Medical Sciences, Tabriz, Iran; 4. Tabriz Health Services Management Research Center, Tabriz University of Medical Sciences, Tabriz, Iran

**Keywords:** Non-communicable diseases, Iranian population, Lifestyle, Intervention

## Abstract

**Background:**

The Lifestyle Promotion Project (LPP) is a long-term community-based project for prevention and control of non-communicable diseases (NCDs). In this project, the healthy lifestyle promotion plan will be implemented by the health policy agenda of East Azerbaijan Province, Iran.

**Methods:**

The study design included two phases: phase I was a cross-sectional prevalence study of NCDs and the associated risk factors implemented from Feb 2014 to Apr 2014. Phase II is a prospective follow-up study currently ongoing. The healthy lifestyle promotion intervention consists of five core strategic plans. These programs was implemented by the health policy agenda of East Azerbaijan Province. Overall, 3000 participants aged 15–65 yr were enrolled to evaluate the impact of healthy lifestyle interventions in phase II of project.

**Results:**

The experience of the LPP in Iran may support the idea that a well-organized, fully evidence-based, and well-developed community-based program could be affordable to prevent non-communicable disorders in developing countries.

**Conclusion:**

The results of this survey will be presented as research articles and reports for policy makers.

## Introduction

Non-communicable diseases (NCD) are the major cause of death worldwide (1). Almost 80% of NCD deaths happen in low and middle-income countries (2). Moreover, based on WHO estimation, NCDs death will increase by 17% over the next ten years. The greatest increase will be seen in the African and the Eastern Mediterranean regions (3). In this context, the Middle East is expected to bear one of the world’s greatest increases in the total burden of NCDs and related risk factors in the upcoming years.

Daily, 800 to 850 deaths occurs in Iran which 89% of them are related to five main reasons: Cardiovascular disease (46%), accidents (17%), cancer (14%), respiratory diseases (6%) and disease around the time of birth (6%) (4). The last survey of risk factors for NCD in East Azerbaijan Province in 2009 showed that approximately 88% of the population consumed less than 5 servings of fruit and vegetables daily (5). Per capita consumption of salt was 2 to 3 fold higher than the world average (6). About 48% of the population had low physical activity and the prevalence of overweight and obesity was about 43.9% (7). About 11% to 12% of population aged 15 to 64 yr are daily smokers, and the prevalence of hypertension was 16% in this population (7).

Interventions to improve lifestyle behaviors and clinical risk factors and prevention of non-communicable diseases in Iran are essential. Hence, the healthy lifestyle program (2015–2025) is designed by the health policy agenda of East Azerbaijan Province.

In this project, the healthy lifestyle promotion plan will be implemented by the health policy agenda of East Azerbaijan Province, Iran. This study is community-based intervention trial for measuring performance of healthy lifestyle promotion plan.

## Methods

### Research goals

The research primary goal was determining the differences on the prevalence of major NCDs risk factors and outcomes before and after healthy lifestyle intervention.

Secondary research goal included an evaluation of the effectiveness of lifestyle promotion interventions in preventing the expansion of NCDs and risk factors by comparing the results with data from other neighboring provinces as a control group.

### Study design

The LPP design had two major components; phase I was a cross-sectional prevalence study of NCDs and its associated risk factors implemented from Feb 2014 to Apr 2014. Phase II (currently ongoing) was a prospective follow-up study began from Feb 2016.

### Ethics approval and consent to participate

The study protocol was approved by Ethics Committee of Tabriz University of Medical Sciences (1394.383) and with the 1964 Helsinki declaration and its later amendments or comparable ethical standards. All subjects were aware of the content of the study and a written informed consent document was obtained. The results of this survey will be presented as research articles and reports for policymakers.

### Study population

Our target population was subjects aged 15 to 64 yr old living in urban and regional areas of East Azerbaijan Province. This study was conducted by probability proportional to size (PPS) multistage stratified cluster sampling through which 150 clusters were selected. In this type of sampling, the selection probability for each component was set to be proportional to its size measure. The sampling frame was based on the postal code setting of the national post office, updated yearly. The clusters were selected in this system based on postal code. Each address in this system was summarized in a 10-digit postal code number. In urban areas, clusters included one to several blocks or parts of blocks. Blocks were usually attached buildings. After determining the cluster start point, enrollment and data collection was started. In each cluster, 20 participants (10 male and 10 female) with the age of 15–65 yr were enrolled (3000 participants). This began from the household at the cluster start point and continued toward the other houses until the required numbers of participants were enrolled. Consecutive households were selected based on the geographical location of buildings to the right-hand side of each building. In this project, adults aged between 15–64 yr were included and adults with severe chronic illness requiring bed rest, physical disability, mental disability and the presence of communication barriers, pregnant women were excluded.

### Baseline measurements

The study was conducted based on the WHO STEPS of risk factors for NCDs. This approach uses different stages of risk factor assessment containing information collection using questionnaires (step 1), physical examinations (step 2), and biomedical assessment, (step 3).

Data were collected based on interviews, physical assessments, and anthropometric measurements by a team of health experts. Research survey and examination teams visited households according to previously arranged appointments.

### Research questionnaire Socio-demographic status

This questionnaire included questions concerning the socio-demographic characteristics, including age, gender, educational level, marriage status, employment status, family size and residential area.

### Angina

The history of any chest pain was assessed using the Persian translated Rose questionnaire. Rose angina had been designed for participants who had chest pain during exertion. Rose questionnaire data are interpreted according to previously published guidelines (8).

### Cigarette smoking status

Different categories of cigarette smoking status were defined according to WHO advice (9). Smokes cigarettes at least once a day was defined as daily smoker; who smokes cigarettes but not every day was known as an occasional smoker; ex-smoker was formerly daily or occasional smoker who presently does not smoke; and never smoked defined as who never smoked previously in the past.

### Physical activity levels

International Physical Activity Questionnaire (IPAQ) was used for determining physical activity levels. The validity of the translated form of this questionnaire was tested in the previous study on Iranian subjects (10). Physical activity levels were also classified into three categories (11).

### Generalized Anxiety Disorder

The Generalized Anxiety Disorder 7-item scale (GAD-7) was completed as a brief measure of generalized anxiety disorder. The GAD-7 is a seven-item standardized tool to measure severity of general anxiety (12).

### Dietary assessment

Dietary assessment was performed by means of quantitative food frequency questionnaires (FFQ) which completed through face-to-face personal interviews by expert dietitians.

### Food security

The short form of the Household Food Security Scale consisting of six questions was used for determining food security. This questionnaire was validated in the local language previously (13).

### Food safety

This questionnaire was consist of two parts: the first part including 18 questions regarding food safety knowledge of and the second part including 13 questions regarding the self-reported practice of participants. The food safety knowledge and practice questionnaire is a modified from other researchers (14, 15).

### Anthropometric measurements

BMI was calculated from height and weight data as kg/m^2^. Body-weight was measured to the nearest 0.1 kg on a Seca digital weighing scale, and a stadiometer was used for measuring height to the nearest 0.1 cm with bare feet. Waist circumference (cm) was measured in duplicate while the subjects were wearing light clothing with an anthropometric tape. Waist circumference was measured at the minimum circumference between the iliac crest and the rib cage and Conicity index was calculated.

### Blood pressure measurement

After a 5 min rest, the blood pressure was measured two times in each arm in the sitting position by a trained nurse using a mercury sphygmomanometer (Richter, Germany). After two measurements, blood pressure was measured a third time if there was a difference of more than 10 mmHg in systolic readings and/or 5 mmHg in diastolic readings, and the two readings with the least difference were documented. Hypertension was defined as SBP≥140 and/or DBP≥90 mmHg or current use of antihypertensive medication for management of hypertension, at the time of interview (16).

### Biochemical measurements

A random subsample of 750 participants were referred to laboratory for blood sampling (in each cluster, 5 participants are randomly enrolled). A blood sample was taken after an overnight fast. FBS, total cholesterol, LDL-C, HDL-C, TG, Hb, Ferritin, serum vitamin D, ALT, AST were measured. Serum LDL-C was calculated by Friedewald equation (17). The first-morning spot urine (SU) sample was collected from first voided morning urine and sodium concentration was measured.

### Lifestyle promotion interventions

The healthy lifestyle promotion plan was implemented by the health policy agenda of East Azerbaijan Province. This plan consisted of five core strategic plans as follows:

1- preparing provincial action plan for prevention and control NCDs and the related risk factors,
2- designing and implementing programs to tackle the burden of NCDs,
3- designing and implementing human resources development and capacity building plan,
4- designing and implementing electronic health record system, and
5- reforming the structure of health care services.

### 1- Preparing provincial action plan for prevention and control of NCDs and the related risk factors

A Multi-sectoral steering committee was formed in October 2013 to guide the formulation of the NCD action plan. A roadmap was developed with the coordination of the sectors. Twelve thematic groups namely: cardiovascular diseases, chronic obstructive lung diseases, cancer, diabetes mellitus, oral health, mental health, road traffic injuries, tobacco, alcohol, unhealthy diet, physical exercise and healthy behavior, air pollution were formed. The groups were assigned to identify gaps and propose actions for their respective thematic areas and the recommendations were synthesized into a draft of action framework. In this action plan, in partnership with relevant organizations in the health field, 3 key cores entitled standardizing and improving the quality of health services, improving lifestyle and promoting health care system in the community were designed and developed to be able to fulfill the objectives.

### 2- Implementing programs to tackle the burden of NCDs

Healthy lifestyle campaigns, integrating the prevention and control of NCDs and associated risk factors program in PHC and self-management support were the main programs.

### a. Designing and implementing healthy lifestyle campaigns

Health campaigns were designed and applied to provide practical advice and health information using different types of media. In this regard, 7 campaigns were implemented entitled increasing physical activity, increasing dairy products consumption, reducing salt, sugar, and oils intake, smoking cessations and anxiety management. The strategies and activities were as follow:

1- Printing, distributing and installing educational resources such as banners, posters, and leaflets.
2- Attaching the health messages in the bills
3- Implementing educational program and training sessions for different target groups such as health care workers, community health volunteers, officials of food procurement, distribution and sale centers, officials of schools’ buffet, housewives and students
4- Reducing oil and salt consumption in boarding schools, school buffets, hospitals and restaurants
5- Increasing dairy products consumption in schools and governmental organizations
6- Applying strong water pipes smoking bans in cafes
7- Holding competitions related to the campaign topics (essay writing, drawing, cartoon, etc.)

### b. Integrating the prevention and control of NCDs and associated risk factors in PHC

**Fig. 1:**
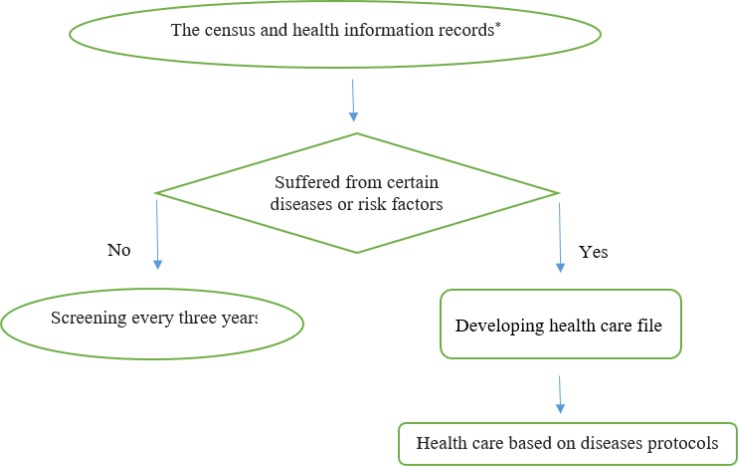
The NCDs care process in the integrated system *Determining dietary behaviors and physical activity levels, determining patients’ history of medication, smoking, alcohol and drugs habit, trauma and injury, measuring blood pressure, weight, height and waist circumference, determining common psychiatric symptoms, determining common symptoms of cardiovascular and respiratory diseases, determining common symptom of cancers (breast, colorectal and cervical cancers), clinical examination based on the protocol

As shown in Table 1, five diseases, five biological risk factors and five behavioral risk factors were considered in this integrated system as “Three 5 program”. Cost-effective and evidence-based interventions and tools to prevent and control various NCDs and risk factors were delivered in an integrated manner through the existing health system. These interventions included the following: decreasing exposure to risk factors through health promotion and primary prevention, early detection and managing, and following to monitor trends in risk factors and diseases. Fig. 1 depicts the NCDs care process in this integrated system.

**Table 1:** NCDs and risk factors considered in the integrated health system

***NCDs***	***Physiological risk factors***	***Behavioral risk factors***
Cardiovascular disease (heart attack and stroke)	High blood pressure	Inappropriate food behavior
Chronic respiratory diseases (asthma and COPD)	Lipid disorders	Risky sexual behavior
Psychiatric disorders (depression and anxiety)	Glucose impaired	Insufficient physical activity
Cancers (breast, cervical and colorectal)	Overweight	Smoking, drinking and taking drugs
Diabetes	Stress	History of trauma and injury

### a. Designing and implementing self-management services

Self-management support was designed to be one of the components of person-centered care in health centers. The role of health care providers in self-management was to act as a consultant, interpreting symptoms, being a resource, offering treatment suggestions and assisting the person in achieving self-management goals. In this project, self-management support was conducted based on the Chronic Care Model. In this model of care, patients and providers worked together to define problems, set priorities, establish goals, create treatment plans, and solve problems as they were encountered.

### 3- Human resources development and capacity building plan

In this plan, enabling the staff to carry out technical, professional and managerial roles effectively were considered. In addition, positions and responsibilities were matched with skills, for example, health facilities and nutrition-specific services were combined. In this newly designed system, nutritional interventions were implemented for people with diseases such as diabetes, over-weight, and obesity, dyslipidemia and hypertension in health centers. The expert dietitian filled out 24-h dietary recall, food habits, and activity forms and provides a diet manual according to each individual’s situation. The dietitian also educated the client about exchange list and how to select foods, manage their diet and self-monitoring skills. In addition, in this system, a mental health professional offered services for improving the mental health of individuals or treating mental illnesses.

### 4. Implementing electronic health record system

This information system was applied for:

1- Managing the collection, storage, processing, retrieval and distribution of service provider information units, including location information, facilities, repairs and related standards in different periods.
2- Managing information of service providers, including roles and access levels.
3- Managing consumers’ information based on existing databases at national and local level and provide health care based on age, sex, danger signs, conditions, place of residence, family size and follow-up through the tools available to service providers
4- Providing periodic records, daily, monthly, seasonal and yearly based on events recorded in the system and categorize the information based on requested events such as Iranian vital horoscope and non-communicable diseases by age and sex.
5- Providing clinical and laboratory services and self-care information as needed.
6- Financial management of the service provider, the amount of credit and the cost of award based on performance.
7- Managing the time and frequency of services for service providers based on characteristics of consumers and monitoring service providers’ performance.
8- Providing a decision support system intended to help **decision** makers compile useful information from

### 5- Reforming the structure of health care services

In this new structure called Health Complex (HC), an accountable and responsive health care system established to deliver integrated care services to people in a defined catchment area against identified per capita payment, under district health center policies and regulations.

Each HC covered a population of 40000 to 120000 in a defined geographic area and included a Comprehensive Health Center (CHC) health centers and a management center which usually located in CHC. The management center was actually the first level of management where health experts (family and school health, diseases, occupational and environmental, logistic and financial support experts) were located in. Based on population density, 2 to 5 health teams (8000 to 16000 people) were classified in one health center. Each health team consisting of a general practitioner and a family health nurse who covered around 4000 people to deliver prevention, promotion, treatment and NCDs care services. One of the physicians was in charge of the center.

CHC was one of the health centers which was suitable and accessible for delivering integrated care services. 24 h emergency and ambulatory services and referral specialist clinic were located in this center. Moreover, in each CHC, a dietitian, a mental health professional and an occupational and environmental expert had covered the services. Health complexes could be administered as public, private, cooperative or a combination of the above.

## Discussion

Chronic diseases are the leading causes of death and disability (18). Lifestyle modification can prevent a large proportion of these diseases (18). It remains a challenge for health experts and governmental systems to alter unhealthy behaviors in populations (19). In practice, the reorientation of health care toward health promotion is a challenging task (20).

Iran suffers from the great burden of NCDs and based on the report of WHO, from the 395000 deaths in Iran, approximately 76% of them have been devoted to NCDs (21). According to this scenario, preventive programs to reduce NCDs risk in the public health system, and low-cost interventions are desired. The new efforts have taken place to prevention and control of the NCDs, especially in East Azerbaijan Province. In order to promote public health and decrease the burden of NCDs, new interventions and policies have been implemented and five core **strategic priorities** have been executed. Many of the current chronic disease management strategies were first identified as the Wagner chronic care model (Wagner CCM), included six key elements (22–24). “These elements focus on mobilizing community resources, promoting high-quality care, enabling patient self-management, implementing care consistent with evidence and patient preferences, effectively using patient/population data, cultural competence, care coordination and health promotion (25)”. Various countries in the world are initiating community-based programs to control non-communicable diseases ( 19, 26, 27). These interventional programs may differ according to the community, socioeconomic, culture and health settings. In all types of interventions, monitoring and evaluation are the most important element of managing health-related intervention programs which resulted in improving the quality of services provided and valuable in finding the strengths and weaknesses of the program. In order to generate an estimate of effectiveness, it is necessary to have a control group with no intervention. However, having a control group in evaluating health promotion interventions has been considered as inappropriate, confusing and unreasonably expensive by a WHO Working Group (28). Feasibility of finding a truly comparable control region and inability to avoid contamination is the main concern.

The main strength of present study is that a large, innovative, multi-site and multi-setting approach for prevention and control of NCDs will be implemented and these plans are evaluated through a large-scale prospective study. The lack of an appropriate control group is a limitation of this project. Thus, caution in interpreting the outcomes needs to be implemented.

## Conclusion

The LPP has been designed in Iran as a prospective study aimed at implementing healthy lifestyle interventions and monitoring the trend of NCD risk factors. The interventional phase of the study is aimed for lifestyle modification of the general population. The experience of the LPP in Iran may support the idea that a well-organized, fully evidence-based, and well-developed community-based program could be affordable to prevent non-communicable disorders in developing countries undergoing nutritional transition.

## Ethical considerations

Ethical issues (Including plagiarism, informed consent, misconduct, data fabrication and/or falsification, double publication and/or submission, redundancy, etc.) have been completely observed by the authors.
